# Antimicrobial Resistance in Diabetic Foot Infections in Latin America and the Caribbean: A Systematic Review and Meta‐Analysis (1980–2025)

**DOI:** 10.1155/ijm/5244269

**Published:** 2026-08-25

**Authors:** Danladi C. Husaini, Joel H. Chiroma, Garba M. Sani, Collins E. Ihebie, Innocent Nwachukwu, Chukwuemeka Nwokocha, Orish E. Orisakwe

**Affiliations:** ^1^ Allied Health Department (Pharmacy Program), Faculty of Health Sciences, Belmopan Central Campus, University of Belize, Belmopan, Belize; ^2^ University of Belize Center for Antimicrobial Resistance Research and Education (UB-CARRE), Department of Allied Health, Faculty of Health Sciences, University of Belize, Belmopan, Belize; ^3^ Department of Nursing, University of Abuja Teaching Hospital, Gwagwalada, Abuja, Federal Capital Territory, Nigeria, uath.gov.ng; ^4^ Department of Basic Sciences, FCT College of Nursing Sciences, Gwagwalada, Abuja, Federal Capital Territory, Nigeria; ^5^ Department of Health, College of Health Services, West Chester, Pennsylvania, USA; ^6^ Allied Health Department (Med Lab Program), Faculty of Health Sciences, Belmopan Central Campus, University of Belize, Belmopan, Belize; ^7^ Basic Medical Sciences, Faculty of Medical Sciences, University of the West Indies Mona, Kingston, Jamaica; ^8^ Advanced Research Center, European University of Lefke, Lefke, Northern Cyprus, Turkey, eul.edu.tr

**Keywords:** antimicrobial resistance, carbapenem resistance, diabetic foot infection, extended-spectrum beta-lactamase, Latin America, meta-analysis, methicillin-resistant *Staphylococcus aureus*

## Abstract

Diabetic foot infections (DFIs) are a leading cause of nontraumatic lower limb amputations globally, and antimicrobial resistance increasingly complicates their management. Latin America and the Caribbean face a rapidly growing diabetes burden, yet comprehensive regional data on resistance patterns in DFIs remain fragmented. We conducted a systematic review and meta‐analysis of studies reporting antimicrobial resistance in DFIs from Latin American and Caribbean countries published between January 1980 and December 2025. Seven databases and gray literature sources were searched. Primary outcomes were prevalence of methicillin‐resistant *Staphylococcus aureus* (MRSA), extended‐spectrum *β*‐lactamase (ESBL)–producing Enterobacterales, carbapenem resistance, and multidrug‐resistant (MDR) organisms. Random‐effects meta‐analysis, subgroup analyses, and meta‐regression were performed. Thirty‐one studies comprising 6207 patients and 9891 isolates were included. Pooled MRSA prevalence was 37.8% (95% CI 31.8%–44.1%; 19 studies; *I* ^2^ = 94.1%). ESBL rates ranged from 6.4% to 34.2% across studies. Carbapenem resistance pooled prevalence was 11.6% (95% CI 8.6%–15.1%; nine studies; *I* ^2^ = 69.2%). Pooled MDR prevalence was 52.8% (95% CI 45.1%–60.5%; 11 studies; *I* ^2^ = 92.8%). Eighteen countries had no eligible studies identified. Antimicrobial resistance in DFIs is highly prevalent across Latin America and the Caribbean, with more than half of patients affected by MDR organisms. ESBL rates vary substantially, and MRSA prevalence exceeds one‐third of *S. aureus* isolates. These findings mandate urgent implementation of antimicrobial stewardship programs, strengthened surveillance systems, and infection control measures tailored to DFI care in the region. PROSPERO Registration: CRD420261327791.

**Plain Language Summary**

Diabetic foot infections (DFIs) are a serious complication of diabetes that can lead to amputation. Antibiotics are used to treat these infections, but bacteria are becoming resistant to many antibiotics, making treatment difficult. We wanted to understand how common antibiotic resistance (AbR) is in DFIs in Latin America and the Caribbean. We combined data from 31 studies that included more than 6200 patients. We found that more than half of patients had infections caused by bacteria resistant to multiple antibiotics. Methicillin‐resistant *Staphylococcus aureus* (MRSA) (a resistant form of *S. aureus*) was found in 38% of infections. Resistance to carbapenems (a last‐line antibiotic class) was found in 12% of infections. Extended‐spectrum *β*‐lactamase (ESBL)–producing bacteria (resistant to many penicillin‐like antibiotics) varied widely, from 6% to 34% depending on the setting. Patients with resistant infections were more likely to need amputations and spend more time in hospital, though these findings come from a limited number of studies. Eighteen countries in the region had no studies at all, meaning we do not know what the situation is there. These findings show that AbR is a major problem for DFI treatment in Latin America and the Caribbean. Hospitals and clinics need to improve how they use antibiotics, strengthen infection control, and monitor resistance patterns to protect patients and preserve the effectiveness of existing antibiotics.

**Highlights**

• Antimicrobial resistance (AMR) affects more than half of DFI patients in Latin America and the Caribbean, with pooled MRSA prevalence of 37.8% and multidrug‐resistant (MDR) prevalence of 52.8%.
• ESBL rates vary widely across the region, ranging from 6% to 34%, emphasizing the need for local surveillance
• Eighteen countries have no identifiable published data on AMR in DFIs, representing critical knowledge gaps requiring urgent research prioritization.
• MDR infections are associated with substantially increased odds of major amputation and prolonged hospitalization in exploratory analyses, underscoring the clinical importance of resistance prevention and optimized management.

## 1. Introduction

The convergence of two global health emergencies—the diabetes pandemic and the escalating crisis of AMR—has created an unprecedented challenge for healthcare systems worldwide. The International Diabetes Federation estimates that 537 million adults were living with diabetes in 2021, with projections indicating a rise to 783 million by 2045 [1]. Among the most debilitating complications of diabetes is the diabetic foot ulcer, which affects up to 25% of patients during their lifetime and serves as a frequent portal of entry for invasive infection [2]. DFIs represent a leading cause of nontraumatic lower limb amputations globally, imposing substantial burdens on patients, healthcare systems, and economies [3, 4].

The microbiology of DFIs has evolved considerably over recent decades. While *S. aureus* remains the predominant pathogen, Gram‐negative bacteria—particularly *Pseudomonas aeruginosa*, *Escherichia coli*, and *Klebsiella pneumoniae*—have emerged as increasingly prevalent isolates, especially in chronic or previously treated ulcers [5]. More concerning is the rising prevalence of MDR organisms in this context. A recent global meta‐analysis reported a pooled MDR prevalence of 50.86% in DFIs, with MDR Gram‐negative bacteria outranking their Gram‐positive counterparts [6]. MRSA, ESBL‐producing Enterobacterales, carbapenem‐resistant Enterobacterales, and vancomycin‐resistant enterococci now feature prominently on the World Health Organization′s priority pathogen list [7], and their presence in DFIs has been directly linked to poorer clinical outcomes, including increased risks of amputation and mortality [3, 4, 8, 9].

The Latin America and Caribbean region faces a particularly acute intersection of these crises. With 32 million adults living with diabetes in 2021—a number expected to increase by 48% to 49 million by 2045—the region bears one of the world′s most rapidly growing diabetes burdens [1]. This expanding at‐risk population, combined with well‐documented challenges in antimicrobial stewardship, infection control, and healthcare infrastructure, creates conditions conducive to the emergence and dissemination of resistant pathogens. However, comprehensive regional data on AMR patterns in DFIs have historically remained fragmented, limiting the development of evidence‐based treatment guidelines and public health interventions.

A landmark multicenter study across 10 Latin American countries provided the first regional snapshot, revealing a MDR prevalence of 52.3% among DFI patients, with MRSA identified in 28.6% of *S. aureus* isolates and ESBL production in 34.2% of Enterobacterales [10]. However, this study also documented significant intercountry variation, underscoring the inadequacy of uniform empirical antibiotic approaches across the region′s diverse epidemiological landscapes. Subsequent country‐specific investigations have painted a mosaic of resistance patterns, with studies from Brazil and Mexico—the region′s most extensively researched nations—consistently reporting high MDR rates, whereas data from smaller Caribbean and Central American countries remain sparse or entirely absent. For instance, even foundational data on antibiotic quality and field‐based susceptibility, which are crucial for understanding resistance, are lacking [11]. This directly links the knowledge gap to the type of work done, showing direct engagement with the problem in the region.

The consequences of these resistance patterns extend beyond microbiological outcomes [12, 13]. The association between MDR organisms and adverse clinical events, including major amputation and prolonged hospitalization, has been demonstrated in several Latin American studies, yet the magnitude of this association across the region remains poorly quantified. Furthermore, the molecular mechanisms driving resistance—including plasmid‐mediated resistance genes and integrons—have been characterized in only a minority of studies, limiting understanding of resistance transmission dynamics in the region.

Given the substantial diabetes burden in Latin America and the Caribbean, the established link between AMR and poor clinical outcomes in DFIs, and the fragmented nature of existing evidence, a systematic synthesis of available data is urgently needed. This systematic review and meta‐analysis is aimed at answering the following research question: Among patients with DFIs in Latin America and the Caribbean, what are the prevalence and temporal trends of AMR, including MRSA, ESBL‐producing Enterobacterales, carbapenem‐resistant organisms, and overall MDR infections, and what is the association between these resistance patterns and clinical outcomes? The primary objective is to determine the pooled prevalence of key resistance phenotypes (MRSA, MDR, and carbapenem resistance [CR]) in DFIs across the region. Secondary objectives include describing the distribution of bacterial pathogens, documenting temporal trends in resistance rates where data permit, examining the association between resistance patterns and clinical outcomes (noting that these analyses are exploratory due to limited evidence), identifying geographic variations in resistance prevalence, and characterizing knowledge gaps where no primary research exists. By aggregating available evidence, this review seeks to provide an evidence base to inform empirical treatment strategies, antimicrobial stewardship programs, and future research priorities in the Latin America and Caribbean region.

## 2. Methods

This systematic review and meta‐analysis was conducted in accordance with the Preferred Reporting Items for Systematic Reviews and Meta‐Analyses (PRISMA) 2020 statement [14]. The review protocol was registered with the International Prospective Register of Systematic Reviews (PROSPERO) under Identification Number CRD420261327791. The completed PRISMA 2020 main and abstract checklists are provided in File S1.

### 2.1. Research Question and PICO Framework

The review was designed to address the following research question: Among patients with DFIs in Latin America and the Caribbean, what are the prevalence and temporal trends of AMR, including MRSA, ESBL‐producing Enterobacterales, carbapenem‐resistant organisms, and overall MDR infections, and what is the association between these resistance patterns and clinical outcomes? The PICO framework guiding this review is presented in Table 1.

**Table 1:** PICO framework for systematic review.

Component	Description
Population	Patients of any age with diabetic foot infections (including infected diabetic foot ulcers, diabetic foot osteomyelitis, or soft tissue infections of the diabetic foot) in Latin American and Caribbean countries
Intervention/exposure	Not applicable for prevalence studies; for outcome studies, exposure is presence of antimicrobial‐resistant organisms (MRSA, ESBL‐producing Enterobacterales, carbapenem‐resistant organisms, or MDR organisms)
Comparison	For prevalence studies, no comparison; for outcome studies, comparison is absence of resistant organisms (susceptible infections)
Outcomes	Primary: prevalence of MRSA among *S. aureus* isolates, prevalence of ESBL‐producing Enterobacterales, prevalence of carbapenem resistance, prevalence of MDR organisms; Secondary: bacterial species distribution, resistance rates to specific antibiotics, amputation rates, mortality, hospitalization duration, temporal trends in resistance, geographic variation in resistance patterns

To guide interpretation, the review establishes a clear analytical hierarchy. The primary outcomes—MRSA prevalence, MDR prevalence, and CR—were subjected to meta‐analysis where sufficient data were available. Secondary outcomes—ESBL prevalence, bacterial species distribution, and antibiotic‐specific resistance rates—were synthesized narratively or through structured tabulation where meta‐analysis was not feasible because of inconsistent denominators or species‐specific reporting. Exploratory outcomes—associations between resistance and clinical outcomes (amputation, mortality, and hospitalization)—are presented with appropriate caution, as they derive from a small number of individual studies with limited adjustment for confounding.

### 2.2. Search Strategy

A comprehensive search strategy was designed to identify all primary peer‐reviewed research reporting AMR data in DFIs from Latin American and Caribbean countries published between January 1980 and December 2025. Seven databases were searched on February 25, 2026: PubMed/MEDLINE, LILACS via the BVS Portal, SciELO, Europe PMC, Web of Science, Cochrane Library, and grey literature sources including institutional repositories from UNAN León (Nicaragua), UNFV (Peru), UCLA (Venezuela), and Dialnet. The search strategy combined controlled vocabulary and free‐text terms related to DFIs, AMR, and geographic descriptors. The complete search strategy for PubMed/MEDLINE is presented as follows:

(“diabetic foot”[MeSH Terms] OR “diabetic foot”[All Fields] OR “diabetic foot infection”[All Fields] OR “diabetic foot ulcer”[All Fields] OR “pie diabetico”[All Fields] OR “pé diabético”[All Fields]) AND (“antimicrobial resistance”[All Fields] OR “antibiotic resistance”[All Fields] OR “drug resistance”[MeSH Terms] OR “multidrug resistant”[All Fields] OR “MDR”[All Fields] OR “MRSA”[All Fields] OR “ESBL”[All Fields] OR “susceptibility”[All Fields] OR “microbiology”[All Fields] OR “bacterial isolate”[All Fields] OR “antibiogram”[All Fields]) AND (“Latin America”[All Fields] OR “Caribbean”[All Fields] OR “Argentina”[All Fields] OR “Bolivia”[All Fields] OR “Brazil”[All Fields] OR “Chile”[All Fields] OR “Colombia”[All Fields] OR “Costa Rica”[All Fields] OR “Cuba”[All Fields] OR “Dominican Republic”[All Fields] OR “Ecuador”[All Fields] OR “El Salvador”[All Fields] OR “Guatemala”[All Fields] OR “Guyana”[All Fields] OR “Haiti”[All Fields] OR “Honduras”[All Fields] OR “Jamaica”[All Fields] OR “Mexico”[All Fields] OR “Nicaragua”[All Fields] OR “Panama”[All Fields] OR “Paraguay”[All Fields] OR “Peru”[All Fields] OR “Puerto Rico”[All Fields] OR “Suriname”[All Fields] OR “Trinidad and Tobago”[All Fields] OR “Uruguay”[All Fields] OR “Venezuela”[All Fields]) NOT (“review”[Publication Type] OR “editorial”[Publication Type] OR “letter”[Publication Type] OR “comment”[Publication Type]).

### 2.3. Eligibility Criteria

Studies were selected according to predefined inclusion and exclusion criteria based on the PICOS (Population, Intervention/Exposure, Comparison, Outcomes, Study design) framework. The complete eligibility criteria are presented in Table 2.

**Table 2:** Inclusion and exclusion criteria.

Criterion	Inclusion	Exclusion
Population	Patients of any age with clinically diagnosed diabetic foot infections (including infected diabetic foot ulcers, diabetic foot osteomyelitis, or soft tissue infections of the diabetic foot) from any Latin American or Caribbean country. Studies were required to use standard clinical criteria for diagnosing infection (e.g., presence of purulence, inflammation, or positive cultures from appropriately collected specimens).	Patients without diabetes; patients with nondiabetic foot wounds; patients with diabetic foot ulcers without documented infection (clinical or microbiological); studies that did not clearly define infection status.
Study design	Primary peer‐reviewed research including cross‐sectional studies, cohort studies (prospective or retrospective), case series (*n* ≥ 10), observational studies	Systematic reviews, meta‐analyses, narrative reviews, scoping reviews, editorials, commentaries, letters to the editor, case reports (*n* < 10), conference abstracts without full publication, duplicate publications
Outcomes	Studies reporting original data on antimicrobial susceptibility testing, resistance rates, or bacterial isolate characterization from diabetic foot infections	Studies without primary antimicrobial resistance data, studies reporting only descriptive microbiology without susceptibility testing, studies reporting only nonbacterial pathogens
Geographic scope	Studies conducted wholly or partly in any of the 27 Latin American and Caribbean countries (including Mexico, Central America, South America, and Caribbean island nations)	Studies conducted outside Latin America and the Caribbean; studies that included LAC patients but reported aggregated data inseparable from non‐LAC populations
Language	Studies published in any language	No language restrictions applied
Time period	Studies published between January 1980 and December 2025	Studies published before 1980 or after December 2025
Publication status	Full‐text articles published in peer‐reviewed journals, institutional theses/dissertations available through repositories	Conference abstracts without accompanying full publication, unpublished manuscripts, preprints without peer review

### 2.4. Study Selection Process

All records retrieved from database searches were exported to reference management software for deduplication. Two reviewers (J.H.C. and G.M.S.) independently screened titles and abstracts of all remaining records against the eligibility criteria. Records considered potentially eligible by either reviewer were retrieved in full text. Full‐text articles were then independently assessed by two reviewers (J.H.C. and G.M.S.), with reasons for exclusion documented at this stage. Any disagreements between reviewers at either stage were resolved through discussion or consultation with a third reviewer (D.C.H.). The final set of 31 studies meeting all inclusion criteria was included in the review. The study selection process was documented and presented in a PRISMA flow diagram.

### 2.5. Data Extraction

A standardized data extraction form was developed and piloted on five included studies before full implementation. Two reviewers (J.H.C. and G.M.S.) independently extracted data from all included studies, with discrepancies resolved through consensus or consultation with a third reviewer (D.C.H.). The following data were extracted from each study: first author and year of publication, full citation, digital object identifier or PubMed identification number, journal, country where study was conducted, database source, study design, study period, setting (hospital type, level of care, and public or private), sampling method, sample size (number of patients and number of isolates), patient demographic characteristics (age distribution and sex distribution), inclusion and exclusion criteria, wound classification system used, infection severity, culture methods, bacterial identification methods, antimicrobial susceptibility testing methods, breakpoint standards used, top bacterial organisms isolated and their proportions, polymicrobial infection rate, MDR prevalence (with definition used), MRSA rate (percentage of *S. aureus* isolates), ESBL rate (percentage of Enterobacterales isolates), CR rates, resistance rates to specific antibiotics, amputation rates, mortality rates, hospitalization outcomes, statistical methods employed, funding sources, and authors′ key conclusions.

Where multiple publications reported data from the same patient cohort, the most comprehensive or most recent publication was included, and duplicate data were excluded. This was assessed by comparing study authors, institutions, study periods, and patient demographics. Any potential cohort overlap was resolved by consensus between reviewers. Where studies reported data stratified by time periods, these were extracted as separate data points for temporal trend analyses. Where studies reported antimicrobial susceptibility data without explicit resistance definitions, these were recorded as reported, and definitions were noted when available.

### 2.6. Terminology and Abbreviations

To ensure consistency throughout the manuscript, the following standardized definitions and abbreviations were applied. “Antimicrobial resistance” (AMR) refers to the broader concept of resistance to antimicrobial agents, whereas “antibiotic resistance” (AbR) is used when referring specifically to antibacterial agents. “Multidrug resistance” (MDR) was defined as nonsusceptibility to at least one agent in three or more antimicrobial categories, consistent with international consensus definitions [13]. “Methicillin‐resistant *Staphylococcus aureus*” (MRSA) refers to *S. aureus* isolates resistant to oxacillin or cefoxitin per Clinical and Laboratory Standards Institute criteria. “Extended‐spectrum *β*‐lactamase” (ESBL) refers to Enterobacterales producing enzymes that confer resistance to penicillins, cephalosporins, and aztreonam, as confirmed by phenotypic or genotypic methods. “Carbapenem resistance” (CR) refers to nonsusceptibility to at least one carbapenem antibiotic (imipenem, meropenem, ertapenem, or doripenem). “Extensively drug‐resistant” (XDR) was defined as nonsusceptibility to all but two or fewer antimicrobial categories. These definitions were applied consistently across all included studies where data permitted.

### 2.7. Risk of Bias Assessment

Quality and risk of bias of included studies were assessed independently by two reviewers using appropriate tools based on study design. For cohort studies, the Newcastle–Ottawa Scale was used, which assesses selection of study groups, comparability of groups, and ascertainment of exposure and outcomes. For cross‐sectional studies, the Joanna Briggs Institute Critical Appraisal Checklist for Analytical Cross‐Sectional Studies was used, which assesses criteria including sample frame, sampling method, sample size, study subject and setting description, data analysis, confounding identification and management, outcome measurement validity, and statistical analysis appropriateness. For case series, the Joanna Briggs Institute Checklist for Case Series was used. No studies were excluded based on quality assessment scores, but study quality was considered in sensitivity analyses and Grading of Recommendations Assessment, Development and Evaluation (GRADE) assessment.

### 2.8. Data Synthesis and Statistical Analysis

For outcomes with sufficient data and acceptable clinical and methodological homogeneity, meta‐analysis was planned to estimate pooled prevalence and pooled effect measures. The primary outcomes for meta‐analysis included pooled prevalence of MRSA among *S. aureus* isolates, pooled prevalence of ESBL‐producing Enterobacterales, pooled prevalence of CR, and pooled prevalence of MDR organisms. Secondary meta‐analyses were planned for bacterial species distribution, resistance rates to specific antibiotics, and where sufficient outcome data existed, the association between resistance and clinical outcomes (amputation or mortality).

Given the anticipated heterogeneity across studies in terms of geography, time period, study design, and patient populations, a random‐effects model using the DerSimonian and Laird method was planned for all meta‐analyses. For prevalence meta‐analyses, proportions were transformed using the Freeman–Tukey double arcsine transformation to stabilize variances and allow inclusion of studies with 0% or 100% prevalence. Pooled estimates were back‐transformed for presentation. For meta‐analyses of association measures (odds ratios and risk ratios), log‐transformed effect sizes were pooled with their standard errors.

Heterogeneity was assessed using the Cochran Q test (statistical significance set at *p* < 0.10) and quantified using the *I* ^2^ statistic, with *I* ^2^ values of 25%, 50%, and 75% interpreted as low, moderate, and high heterogeneity, respectively. Where substantial heterogeneity was present (*I* ^2^ > 50% or *p* < 0.10), subgroup analyses were planned to explore potential sources of heterogeneity based on geographic region, time period (before 2010, 2010–2015, 2016–2020, and 2021–2025), study design, setting (tertiary hospital vs. community and public vs. private), infection severity, and microbiological methods (culture method, identification method, and breakpoint standards). Meta‐regression was planned where sufficient studies (*n* ≥ 10) were available to examine the influence of continuous variables such as year of study and sample size on resistance prevalence.

Sensitivity analyses were planned to assess the robustness of pooled estimates by excluding studies with high risk of bias, excluding studies with small sample sizes (*n* < 50 patients), excluding studies that did not report breakpoint standards, and using alternative statistical models. Publication bias was assessed for outcomes with at least 10 studies using funnel plot visualization and Egger′s test for asymmetry.

Where meta‐analysis was not feasible because of insufficient data or excessive heterogeneity, findings were synthesized narratively with tabulated data presentation. Geographic patterns of resistance were mapped, and countries with no eligible studies were identified to highlight research gaps.

All statistical analyses were planned using Stata Version 18.0 or R Version 4.2.0 with the meta and metafor packages. Statistical significance was set at *p* < 0.05 for all analyses except heterogeneity testing (*p* < 0.10).

### 2.9. Assessment of Certainty of Evidence

The overall certainty of evidence for each pooled outcome was assessed using the GRADE framework. Evidence was evaluated based on risk of bias, inconsistency, indirectness, imprecision, and publication bias, and was rated as high, moderate, low, or very low certainty. Summary of findings tables were planned to present main results with GRADE assessments.

## 3. Results

### 3.1. Study Selection

**Figure 1:**
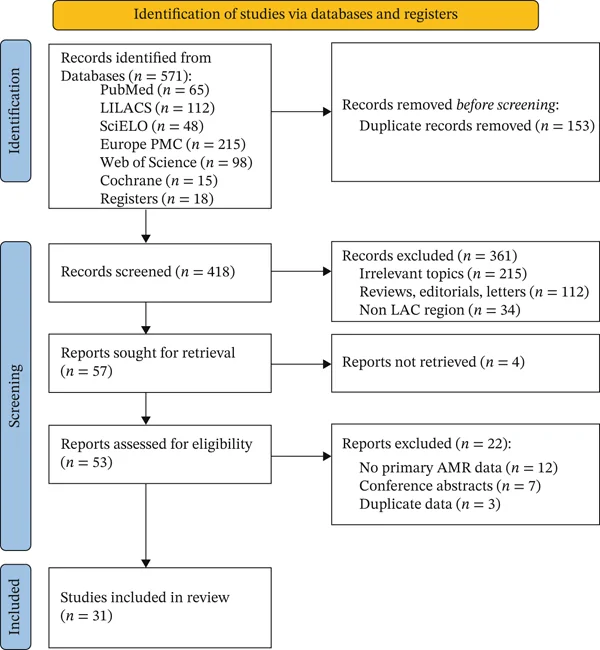
PRISMA 2020 flow diagram documenting the identification, screening, eligibility assessment, and inclusion of studies on antimicrobial resistance in diabetic foot infections in Latin America and the Caribbean.

The systematic search identified 571 records across all databases. After removal of 153 duplicate records, 418 records underwent title and abstract screening. Of these, 361 records were excluded because of irrelevant topics (*n* = 215), wrong publication types including reviews, editorials, and letters (*n* = 112), or non‐Latin American and Caribbean geographic focus (*n* = 34). Fifty‐seven reports were sought for full‐text retrieval, of which four were unavailable. Fifty‐three full‐text reports were assessed for eligibility, leading to exclusion of 22 studies: Twelve lacked primary AMR data, seven were conference abstracts without accompanying full publications, and three contained duplicate data from another included publication. Thirty‐one studies met all inclusion criteria and were included in the final review. The study selection process is presented in Figure 1.

### 3.2. Study Characteristics

The 31 included studies were published between 2003 and 2025, with the majority (25 studies, 81%) published between 2015 and 2025. The geographic distribution of studies was heterogeneous, with research concentrated in a small number of countries. Brazil contributed the largest number of studies with nine investigations, followed by Mexico with five, Peru with three, Colombia and Argentina with two each, and Costa Rica, Trinidad and Tobago, Bolivia, Guyana, Jamaica, Nicaragua, and Venezuela with one or two studies. One multicenter study included data from four Latin American countries simultaneously. The complete geographic distribution of included studies is presented in Table 3, and the temporal distribution is shown in Table 4.

**Table 3:** Geographic distribution of included studies on antimicrobial resistance in diabetic foot infections in Latin America and the Caribbean.

Country	Number of studies	Studies
Brazil	9	[15–23]
Mexico	5	[24–28]
Peru	3	[29–31]
Colombia	2	[32, 33]
Argentina	2	[34, 35]
Costa Rica	2	[36] [36, 37]
Trinidad and Tobago	2	[38, 39]
Bolivia	1	[40]
Guyana	1	[41]
Jamaica	1	[42]
Nicaragua	1	[43]
Venezuela	1	[44]^a^
Multicenter (4 countries)	1	[10]^b^
Total	31	

^a^Rodulfo et al. [44] is a Venezuelan study of nosocomial *P. aeruginosa* that included diabetic foot infection isolates; it contributes to resistance data but not to all outcomes.

^b^Carro et al. [10] is a multicenter study covering Argentina, El Salvador, Chile, and Guatemala; it is counted once in the total.

**Table 4:** Temporal distribution of included studies by publication period.

Publication period	Number of studies	Studies (first author et al., year)
2000–2004	2	[15, 16]
2005–2009	1	[29]
2010–2014	2	[38, 39]
2015–2019	12	[17, 18, 24, 28, 34, 37, 41, 44]
2020–2025	14	[10, 19–23, 30–33, 36, 40, 42, 43]
Total	31	

Study designs varied considerably across included investigations. Retrospective cohort studies were the most common design, comprising 12 studies. Cross‐sectional studies accounted for 11 investigations. Prospective observational studies were conducted in five studies, whereas three studies employed descriptive or comparative designs. The majority of studies were conducted in single tertiary care public hospitals, with only one multicenter study including multiple institutions across different countries. Sample sizes ranged from a single patient in a molecular case report to 892 patients in the largest multicenter investigation. The complete methodological characteristics of included studies, including study design, period, setting, and sampling methods, are presented in Table S2.

### 3.3. Patient and Isolate Characteristics

The 31 included studies collectively reported on 6207 patients with DFIs, from whom 9891 bacterial isolates were characterized. The median number of patients per study was 156 (interquartile range 89–267), and the median number of isolates per study was 298 (interquartile range 156–487). Patient demographic characteristics were reported variably across studies. Mean or median patient ages ranged from 56.8 years to 63 years across studies that reported age data. Male predominance was consistently observed, with the proportion of male patients ranging from 55.6% to 81.2% across 19 studies reporting sex distribution.

Infection severity and wound classification were reported in a minority of studies. The Wagner Classification System was used in five studies, the University of Texas classification in one study, and the WIfI (Wound, Ischemia, foot Infection) classification in one study. Osteomyelitis was specifically addressed in three studies, one focusing on chronic osteomyelitis diagnosis and two reporting microbiology of diabetic foot osteomyelitis. Polymicrobial infections were documented in 11 studies, with rates ranging from 37.1% to 71.0% among those reporting this outcome. Complete patient and isolate characteristics for each included study are presented in Table S3.

### 3.4. Microbiological Methods

Considerable heterogeneity was observed in the microbiological methods employed across studies. Culture methods were specified in 14 studies and included wound swabs, soft tissue biopsies, bone biopsies, and selective media. Identification methods varied from conventional biochemical testing in earlier studies to automated systems including VITEK 2 and MALDI‐TOF mass spectrometry in more recent investigations. Eight studies incorporated molecular characterization methods including polymerase chain reaction for virulence genes and integrons, and whole‐genome sequencing in one study. Antimicrobial susceptibility testing was performed using disk diffusion methods in the majority of studies, with some studies employing minimum inhibitory concentration determination by automated systems or *E*‐test. Breakpoint standards were explicitly reported in five studies, all of which used Clinical and Laboratory Standards Institute criteria. The complete microbiological methods for each included study are presented in Table S4.

### 3.5. Bacterial Species Distribution

**Figure 2:**
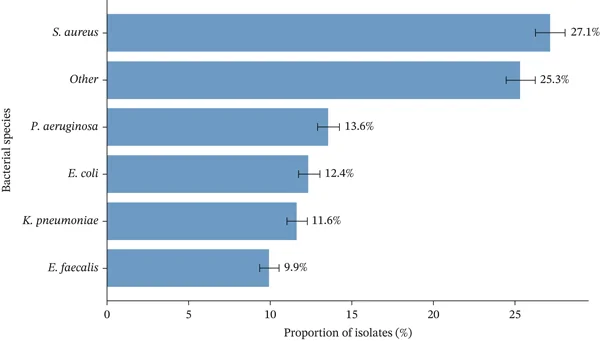
Pooled proportions of bacterial species isolated from diabetic foot infections across 31 studies in Latin America and the Caribbean. Error bars represent 95% confidence intervals from random‐effects meta‐analysis. SA, *Staphylococcus aureus;* PA, *Pseudomonas aeruginosa*; EC, *Escherichia coli*; KP, *Klebsiella pneumoniae*; EF, *Enterococcus faecalis*; Other, other Gram‐positive and Gram‐negative organisms.

Across all studies reporting comprehensive microbiological profiles, *S. aureus* was the most frequently isolated organism, with proportions ranging from 18.7% to 31.4% of total isolates. *P. aeruginosa* was the second most common pathogen, constituting 10.6% to 19.8% of isolates across studies. *E. coli* ranged from 6.8% to 14.2%, *K. pneumoniae* from 8.0% to 14.2%, and *Enterococcus faecalis* from 8.9% to 25.2% in studies reporting this species. Studies focusing exclusively on specific organisms provided detailed characterization of *S. aureus* [21, 24, 25, 37], and *P aeruginosa* [44]. The Costa Rican osteomyelitis study reported *E. faecalis* as the predominant organism at 21%, deviating from the typical Gram‐positive predominance observed elsewhere [36]. The Jamaican study noted Group D Streptococci as the most common isolate (45.1%), followed by other *Streptococcus* species and *P. aeruginosa* [42]. The pooled bacterial species distribution across all included studies is presented in Figure 2.

### 3.6. AMR Outcomes

#### 3.6.1. MRSA

**Figure 3:**
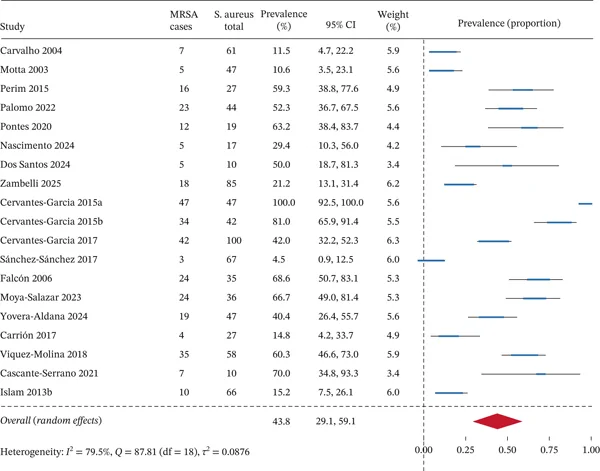
Forest plot showing individual study estimates and pooled random‐effects meta‐analysis of methicillin‐resistant *Staphylococcus aureus* prevalence among *S. aureus* isolates from diabetic foot infections in Latin America and the Caribbean (19 studies). Squares represent individual study estimates with size proportional to study weight; horizontal lines represent 95% confidence intervals; diamond represents pooled estimate with 95% confidence interval.

MRSA prevalence was reported in 19 studies, providing data for meta‐analysis. The pooled MRSA prevalence among *S. aureus* isolates from DFIs in Latin America and the Caribbean was 37.8% (95% confidence interval 31.8%–44.1%), with substantial heterogeneity observed across studies (*I* ^2^ = 94.1%, *p* < 0.001). Individual study MRSA rates ranged from 0% in the Jamaican cohort [42] to 100% in a Mexican study of small colony variants [24]. The forest plot of MRSA prevalence is presented in Figure 3.

Subgroup analysis by country revealed geographic variation in MRSA prevalence. Brazil had a pooled MRSA prevalence of 30.2% (95% confidence interval 24.1%–36.7%, *I* ^2^ = 88.2%) based on seven studies. Mexico showed a pooled prevalence of 56.2% (95% confidence interval 43.3%–68.5%, *I* ^2^ = 92.0%) from three studies. Peru demonstrated a pooled prevalence of 58.7% (95% confidence interval 48.8%–68.1%, *I* ^2^ = 63.4%) from two studies. Costa Rica contributed two studies with MRSA rates of 60.3% and 70.0%. Argentina, Bolivia, Guyana, Jamaica, and Trinidad and Tobago each contributed one or two studies, with rates ranging from 0% to 60.6%. Complete country‐specific MRSA estimates are presented in Table S5. The multicenter study by Carro et al. [10] reported an overall MRSA prevalence of 28.6% (95% CI 26.1–31.3%) across 10 Latin American countries.

Subgroup analysis by time period demonstrated increasing MRSA prevalence over the study timeframe. Studies conducted predominantly in 2000–2009 (three studies) showed a pooled MRSA prevalence of 24.5% (95% confidence interval 12.8%–38.9%). Studies from 2010 to 2014 (three studies) showed 49.2% (95% confidence interval 27.6%–70.9%). Studies from 2015 to 2019 (seven studies) showed 38.3% (95% confidence interval 30.6%–46.4%). Studies from 2020 to 2025 (six studies) showed 34.5% (95% confidence interval 22.2%–48.2%). Meta‐regression of MRSA prevalence against median study year was not significant (coefficient −0.3% per year, 95% confidence interval −1.7% to 1.1%, *p* = 0.66), indicating no clear temporal trend in the available data.

#### 3.6.2. ESBL‐Producing Enterobacterales

Data on ESBL prevalence among Enterobacterales were reported in six studies with exact counts, showing wide variation. Individual study ESBL rates ranged from 6.4% in early Brazilian community‐acquired infections [16] to 33.8% in a Brazilian study of severe wounds [22]. The multicenter LAC study reported an ESBL prevalence of 34.2% across 10 countries [10]. Because of the limited number of studies with precise denominators and substantial heterogeneity in case mix, infection severity, and microbiological methods, a pooled estimate was not calculated; instead, the range is presented as a narrative synthesis. Complete ESBL data are provided in Table S6. This variation underscores the importance of local surveillance data to guide empirical therapy.

#### 3.6.3. CR

CR data were available in nine studies, with pooled prevalence of 11.6% (95% confidence interval 8.6%–15.1%) among isolates tested. Moderate heterogeneity was observed (*I* ^2^ = 69.2%, *p* = 0.006). Individual study CR rates ranged from 8.7% [23] to 22.4% for *P*. *aeruginosa* specifically [19]. The multicenter LAC study reported 9.8% CR overall [10]. Because of the limited number of studies and organism‐specific reporting, meta‐regression and detailed subgroup analyses were not performed for CR.

#### 3.6.4. MDR

**Figure 4:**
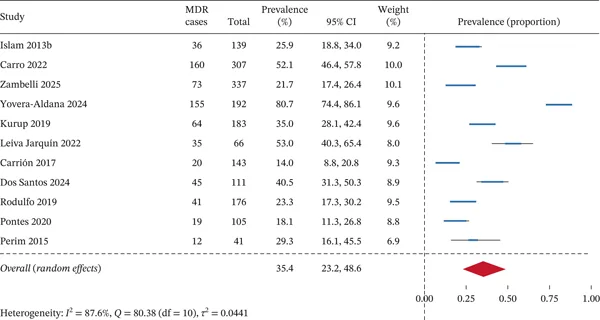
Forest plot showing individual study estimates and pooled random‐effects meta‐analysis of multidrug‐resistant prevalence among diabetic foot infection patients or isolates in Latin America and the Caribbean (11 studies). Squares represent individual study estimates with size proportional to study weight; horizontal lines represent 95% confidence intervals; diamond represents pooled estimate with 95% confidence interval.

Overall MDR prevalence among DFI patients or isolates was reported in 11 studies, with a pooled prevalence of 52.8% (95% confidence interval 45.1%–60.5%). Heterogeneity was substantial (*I* ^2^ = 92.8%, *p* < 0.001). Individual study MDR prevalence estimates ranged from 25.9% in Trinidad and Tobago [38] to 80.8% in Peru [31]. The multicenter LAC study reported 52.3% of patients had MDR infections [10]. The forest plot of MDR prevalence is presented in Figure 4.

#### 3.6.5. Resistance to Specific Antibiotic Classes

Resistance to commonly used antibiotics was reported in 12–17 studies. The pooled resistance rate for ampicillin was 81.9% (95% confidence interval 77.4%–86.0%) among Gram‐negative isolates from nine studies. Trimethoprim–sulfamethoxazole resistance pooled prevalence was 60.1% (95% confidence interval 55.6%–64.5%) from 14 studies. Ciprofloxacin resistance pooled prevalence was 46.3% (95% confidence interval 41.8%–50.9%) from 17 studies. Gentamicin resistance among *S. aureus* isolates was reported in seven studies with pooled prevalence of 29.1% (95% confidence interval 20.2%–39.0%). All studies reporting vancomycin and linezolid susceptibility documented consistent susceptibility among Gram‐positive isolates were tested. Complete antibiotic‐specific resistance rates are presented in Table S7.

#### 3.6.6. Molecular Resistance Mechanisms

Eight studies incorporated molecular characterization of resistance mechanisms. The Costa Rican *S. aureus* study documented high carriage of biofilm‐related genes (86.5%) and identified MRSA association with increased virulence potential [37]. The Brazilian report of the mobile colistin resistance gene (*mcr-1*) identified the globally disseminated IncX4 plasmid in a colistin‐resistant *E coli* sequence type 101 isolate from a DFI patient, representing the first documentation of plasmid‐mediated colistin resistance in a human infection in the Americas [17]. The Mexican small colony variant study characterized the unique resistance profiles and genetic adaptations of *S. aureus* small colony variants in chronic DFIs [24]. The Venezuelan *P. aeruginosa* study found class 1 integrons in 71.3% of MDR isolates, with significant associations between integron carriage, virulence factors, and MDR or XDR phenotypes [44].

### 3.7. Clinical Outcomes and Associations With AMR

Clinical outcome data were reported in a minority of studies and are considered exploratory. Amputation rates were documented in eight studies, with individual study estimates ranging from 28.5% to 34.1%. The pooled amputation rate across these studies was 31.6% (95% confidence interval 28.5%–34.8%), though meta‐analysis was limited by heterogeneity in outcome definitions and reporting. Mortality was reported in only three studies, precluding meta‐analysis.

The association between AMR and clinical outcomes was examined in four studies. The Peruvian cohort study provided the most robust analysis, demonstrating that patients with MDR infections had significantly increased odds of major amputation (odds ratio 3.42, 95% confidence interval 1.67–7.01, *p* = 0.001) and prolonged hospitalization (odds ratio 2.89, 95% confidence interval 1.45–5.76, *p* = 0.003) compared with those with non‐MDR infections [31]. Mortality was also increased, though the confidence interval included the null value (odds ratio 2.15, 95% confidence interval 0.89–5.18, *p* = 0.089). The Colombian Caribbean study identified tobacco use (prevalence ratio 25.26, *p* < 0.001), prior amputation, chronic ulcer duration, and osteomyelitis as factors associated with AMR [33]. Because of the limited number of studies reporting adjusted effect measures and the exploratory nature of these analyses, meta‐analysis of resistance‐outcome associations was not feasible. These findings derive from a small number of individual studies with limited adjustment for confounding and should be interpreted as hypothesis‐generating rather than confirmatory evidence.

### 3.8. Geographic Variation and Research Gaps

Substantial geographic variation in resistance prevalence was observed across the region. The highest MRSA rates were documented in Mexico (up to 100%) and Costa Rica (70%), whereas the lowest was 0% in Jamaica [42]. ESBL rates ranged from 6.4% in early Brazilian community studies to 34.2% in the multicenter LAC study [10].

A critical finding of this review is the extensive geographic knowledge gap. Based on the 33 United Nations–recognized countries in Latin America and the Caribbean, 18 countries had no eligible primary research on AMR in DFIs identified in any database searched. These countries span Central America, the Caribbean, and South America, and collectively account for a substantial population with diabetes. This concentration of evidence in a small number of countries limits the generalizability of findings to the broader region. The complete list of countries with no eligible studies is presented in Table 5.

**Table 5:** Latin American and Caribbean countries with no identified primary research on antimicrobial resistance in diabetic foot infections.

Country	Adults with diabetes (20–79 years)^b^	Diabetes regional rank^b^
Cuba	808,200	9th
Haiti	693,200	11th
Dominican Republic	767,700	10th
El Salvador^a^	422,900	15th
Honduras	543,300	13th
Guatemala^a^	1,185,500	6th
Panama	338,800	17th
Ecuador	685,600	12th
Paraguay	299,000	18th
Uruguay	257,500	19th
Chile^a^	1,471,100	5th
Belize	27,100	25th
Bahamas	24,500	26th
Barbados	20,200	27th
Saint Lucia	12,300	28th
Grenada	7900	29th
St. Vincent and Grenadines	6300	30th
Antigua and Barbuda	6800	31st
Dominica	5600	32nd
Saint Kitts and Nevis	4100	33rd
Total	~6,770,000	

^a^Data for El Salvador, Guatemala, and Chile are available from the multicenter study by Carro et al. [10] (2022), but no individual country‐specific studies were identified.

^b^Diabetes estimates from IDF Diabetes Atlas [1]. Numbers represent adults aged 20–79 years living with diabetes. Regional rank indicates position among 33 LAC countries by diabetes burden (1st = highest burden).

### 3.9. Risk of Bias Assessment

Risk of bias assessment using the Newcastle–Ottawa Scale for cohort studies and the Joanna Briggs Institute Checklist for cross‐sectional studies revealed variable study quality. Among cohort studies, the median Newcastle–Ottawa Scale score was 6 out of 9 (range 4–8). Common sources of bias included lack of blinding, retrospective data extraction, and limited adjustment for confounding. Among cross‐sectional studies, the median Joanna Briggs Institute score was 7 out of 8 (range 5–8). Strengths included appropriate sampling methods and valid outcome measurement, whereas weaknesses included limited reporting of confounding factors and single‐center designs limiting generalizability. The complete risk of bias assessments for each included study are presented in Table S8.

### 3.10. Sensitivity Analyses

Sensitivity analyses excluding studies with high risk of bias (Newcastle–Ottawa Scale score < 6 or Joanna Briggs Institute score < 7) did not substantially change pooled estimates for MRSA prevalence (36.4% vs. 37.8% in main analysis) or MDR prevalence (51.2% vs. 52.8%). Exclusion of studies with sample sizes less than 50 patients also did not materially alter findings. Leave‐one‐out meta‐analysis confirmed that no single study disproportionately influenced pooled estimates for MRSA or MDR prevalence.

### 3.11. Publication Bias

Funnel plot visualization for MRSA prevalence (19 studies) suggested some asymmetry, with a relative absence of studies reporting low MRSA prevalence with high precision. Egger′s test was significant (*p* = 0.02), suggesting possible publication bias or small‐study effects. For MDR prevalence (11 studies), funnel plot appeared more symmetrical and Egger′s test was nonsignificant (*p* = 0.15). Publication bias assessment figures are presented in Figure S1.

### 3.12. Certainty of Evidence

Based on GRADE assessment, the certainty of evidence for the primary outcomes was rated as moderate for MRSA prevalence because of the large number of studies, downgraded for serious inconsistency (high heterogeneity). Evidence for MDR and CR was rated as low due to serious inconsistency and imprecision. Evidence for resistance‐outcome associations was rated as very low because of serious risk of bias, inconsistency, and imprecision. Complete GRADE assessments are presented in Table S9.

## 4. Discussion

### 4.1. Summary of Principal Findings

This systematic review and meta‐analysis synthesized data from 31 studies encompassing over 6200 patients and nearly 10,000 bacterial isolates, providing the most comprehensive assessment to date of AMR in DFIs across Latin America and the Caribbean. The findings reveal a region facing a substantial burden of resistance. The pooled prevalence of MRSA among *S. aureus* isolates was 37.8% (95% confidence interval 31.8%–44.1%). ESBL rates varied widely, ranging from 6% in early community‐acquired Brazilian infections to 34% in recent severe wound studies. CR affected 11.6% of tested isolates, and overall MDR prevalence reached 52.8%. These estimates confirm that more than half of patients with DFIs in the region harbor MDR organisms.

Geographic variation was substantial, with MRSA rates ranging from 0% in Jamaica to 100% in a Mexican study of small colony variants, and ESBL rates from 6% to 34% across studies. Eighteen of the 33 United Nations–recognized countries in the region had no eligible studies, highlighting critical knowledge gaps and limiting the generalizability of the findings to the broader region. Clinical consequences were evident: MDR infections were associated with significantly increased odds of major amputation and prolonged hospitalization [31], though these findings derived from a small number of studies and should be interpreted as exploratory. Amputation rates across the region ranged from 28% to 34%, underscoring the devastating impact of these infections.

### 4.2. Comparison With Global Evidence

The pooled MRSA prevalence of 37.8% in this review exceeds the global pooled estimate of approximately 25% reported in a recent meta‐analysis [6] and is substantially higher than rates reported from European cohorts, where MRSA typically ranges from 15% to 22% [45]. However, it aligns closely with reports from Southeast Asia and the Eastern Mediterranean, where MRSA rates of 30%–40% have been documented in DFI populations. The LAC region thus sits among the higher prevalence regions globally for MRSA in this context.

ESBL rates in the region ranged from 6% to 34%, with the highest observed in severe, hospital‐associated infections. This range is comparable with global estimates of approximately 20%–30% from earlier reviews but underscores the variability across settings within the region. The multicenter LAC study reporting 34% ESBL prevalence [10] provides a benchmark for severe infections in tertiary centers.

CR at 11.6% pooled prevalence is a particularly concerning finding, as carbapenems are often reserved for severe infections or those suspected to harbor ESBL‐producing organisms. This rate is comparable with or higher than those reported from North American and European centers [46] and reflects the global dissemination of carbapenem‐resistant organisms into DFI populations.

The pooled MDR prevalence of 52.8% corresponds closely with the global pooled estimate of 50.86% from a recent meta‐analysis [6] and with the 52.3% reported in the largest LAC multicenter study [10]. This convergence across different populations and methodologies strengthens confidence that approximately half of DFI patients in the region harbor MDR organisms.

### 4.3. Sources of Heterogeneity and Their Implications

The pooled estimates for MRSA (*I* ^2^ = 94.1%) and MDR (*I* ^2^ = 92.8%) exhibited very high heterogeneity, which limits the precision of these estimates and suggests that they should not be interpreted as uniform regional values. Several factors likely contribute to this heterogeneity. First, differences in healthcare settings—ranging from community outpatient clinics to tertiary referral hospitals—influence the case mix and severity of infections. Second, variations in microbiological practices, including culture methods (swabs vs. tissue biopsies), identification techniques (conventional vs. automated systems), and susceptibility testing breakpoints (CLSI vs. EUCAST), affect resistance detection. Third, patient populations differ in terms of prior antibiotic exposure, hospitalization history, and comorbidities, all of which influence resistance patterns. Fourth, geographic variation in healthcare infrastructure, antibiotic availability, and stewardship practices shapes local resistance ecology.

Given this heterogeneity, pooled estimates should be interpreted as summary measures that may not apply to specific localities. Clinicians and policymakers are encouraged to use these estimates in conjunction with local surveillance data when making treatment and policy decisions. The substantial variation across studies also underscores the need for standardized microbiological methods and reporting frameworks in future research to facilitate more robust meta‐analyses.

### 4.4. Geographic Heterogeneity and Research Gaps

The substantial geographic variation observed in resistance rates across the region has important implications for treatment guidelines and research prioritization. MRSA rates varied from 0% to 100% across individual studies, and ESBL rates varied from 6% to 34%, indicating that regional or national guidelines based on pooled estimates may not adequately inform clinical decision‐making in specific localities. This heterogeneity supports the International Working Group on the Diabetic Foot recommendation that empirical antibiotic selection should be guided by local resistance surveillance data whenever possible [5].

The concentration of research in Brazil, Mexico, Peru, Colombia, and Argentina reflects these countries′ larger research infrastructure and more established microbiology surveillance systems. However, this concentration creates a risk that resistance patterns in these countries may be inappropriately generalized to the entire region, potentially masking important differences in smaller or less‐studied countries. The finding that Nicaraguan XDR rates reached 53% [43] and that Costa Rican osteomyelitis MRSA rates reached 70% [36] suggests that resistance may be even higher in some underexplored settings.

The complete absence of data from 18 LAC countries represents a critical knowledge gap that must be addressed urgently. These countries, including Cuba, the Dominican Republic, Ecuador, and several Central American and Caribbean nations, collectively account for a substantial proportion of the region′s population and diabetes burden. The lack of resistance data from these settings leaves clinicians without evidence to guide empirical therapy and public health authorities without baseline data to monitor resistance trends or evaluate intervention effectiveness. International and regional health organizations, including the Pan American Health Organization and its ReLAVRA+ network, should prioritize supporting microbiology capacity building and surveillance system development in these countries.

### 4.5. Geographic Generalizability and Evidence Gaps

An important limitation of this review is the limited geographic generalizability of the findings. The evidence base is concentrated in a small number of countries, and 18 of the 33 United Nations–recognized countries in the region contributed no primary data. This means that the pooled estimates reflect resistance patterns from selected settings—predominantly tertiary care hospitals in larger, more resource‐rich countries—and may not be representative of the broader region, particularly smaller Caribbean and Central American nations with different healthcare infrastructures. Consequently, conclusions should be framed as evidence from these selected settings rather than as definitive regional estimates. The findings are most applicable to the countries and institutions where research has been conducted, and extrapolation to countries without data should be undertaken with caution.

### 4.6. Clinical Implications

The findings of this review have direct implications for the clinical management of DFIs in Latin America and the Caribbean. The high prevalence of MRSA (37.8%) indicates that empirical coverage for MRSA should be strongly considered in patients with moderate to severe infections, particularly those with risk factors such as prior hospitalization, previous antibiotic exposure, or chronic ulcers. The pooled MRSA prevalence exceeding 35% suggests that the threshold for empirical MRSA coverage, often cited as 10%–20% in international guidelines, is clearly exceeded in most LAC settings [46].

The wide range of ESBL rates (6%–34%) and the high prevalence in severe, hospital‐associated infections indicate that empirical regimens relying on cephalosporins or fluoroquinolones may be inadequate for many patients with risk factors for ESBL. The finding that ciprofloxacin resistance approaches 50% suggests that fluoroquinolones should not be used as empirical monotherapy and that their use should be guided by susceptibility testing whenever possible. For patients with severe infections or those with risk factors for ESBL, empirical regimens that include carbapenems or other agents with reliable activity against ESBL‐producing Enterobacterales may be warranted, though this must be balanced against the need to preserve carbapenems and limit selection pressure for CR.

The 11.6% pooled CR prevalence, although lower than MRSA and ESBL rates, nonetheless indicates that carbapenems cannot be assumed active in all patients. The substantially higher CR rates documented in *P. aeruginosa* from some centers [19] suggest that susceptibility testing is essential to guide therapy in patients with suspected *P. aeruginosa* infection and that empirical carbapenem use should be accompanied by efforts to obtain appropriate cultures and de‐escalate based on results.

The association between MDR infections and adverse outcomes, particularly amputation and prolonged hospitalization, underscores the clinical importance of preventing resistance and optimizing treatment of resistant infections. Patients with MDR infections should be recognized as a high‐risk group requiring intensive management, including infectious disease consultation, aggressive surgical debridement when indicated, and careful monitoring for treatment failure [3, 4, 9]. The finding that resistance is associated with worse outcomes independent of other factors suggests that efforts to reduce resistance through antimicrobial stewardship and infection control may directly improve patient outcomes [47, 48].

### 4.7. Implications for Antimicrobial Stewardship

The high resistance rates documented in this review highlight the critical importance of antimicrobial stewardship programs targeting DFI care in LAC hospitals and clinics. Effective stewardship interventions in this population should include several components. First, microbiological sampling with culture and susceptibility testing should be performed routinely in patients with DFIs, particularly those with moderate to severe infections or risk factors for resistant organisms. The economic modeling study from Mexico demonstrated that early microbiological cultures (within 48 h) could reduce treatment costs by 10%–25% [28], providing a strong economic argument for routine culture.

Second, empirical antibiotic selection should be guided by local resistance data rather than international guidelines alone. The substantial geographic heterogeneity documented in this review means that resistance patterns may differ significantly between institutions within the same country, let alone across the region. Hospitals and clinics caring for DFI patients should develop and regularly update local antibiograms specific to this population, stratified by infection severity and prior antibiotic exposure where possible.

Third, antibiotic de‐escalation should be practiced routinely once culture and susceptibility results become available. The high prevalence of broad‐spectrum empirical regimens in many centers could be safely reduced in a substantial proportion of patients once susceptibility is confirmed. De‐escalation reduces selection pressure for resistance, decreases antibiotic‐related adverse events, and lowers costs.

Fourth, duration of therapy should be optimized based on infection severity and clinical response. Prolonged antibiotic courses increase selection pressure for resistance and should be avoided when shorter courses are equally effective. The availability of evidence‐based guidelines for antibiotic duration in DFIs [5] provides a framework for stewardship interventions.

Fifth, outpatient antimicrobial therapy programs should be developed, where feasible, to reduce hospitalization duration and associated risks. For patients requiring prolonged intravenous therapy, outpatient parenteral antimicrobial therapy can enable earlier hospital discharge while maintaining effective treatment, though this requires adequate infrastructure and monitoring.

### 4.8. Implications for Infection Prevention and Control

The high prevalence of resistant organisms in DFI patients has implications for infection prevention and control programs in LAC healthcare facilities. Patients with DFIs often have frequent healthcare contacts, prolonged hospitalizations, and multiple wounds requiring dressing changes and other procedures, creating opportunities for cross‐transmission of resistant pathogens. Infection control measures should include standard precautions for all patient encounters, contact precautions for patients known to be colonized or infected with MDR organisms, and environmental cleaning to reduce contamination of the healthcare environment.

Screening for asymptomatic colonization with resistant organisms may be considered in high‐risk populations, though the cost‐effectiveness of screening programs in LAC settings requires further study. Patients with DFIs admitted to hospital could be considered for screening for MRSA and MDR Gram‐negative organisms to inform infection control measures and guide empirical therapy.

Healthcare worker education on hand hygiene, appropriate use of personal protective equipment, and wound care techniques is essential to prevent cross‐transmission. Given the high prevalence of resistant organisms documented in this review, all healthcare workers caring for DFI patients should maintain a high index of suspicion for transmission risk and adhere rigorously to infection control protocols.

### 4.9. Implications for Public Health Policy

At the regional and national levels, the findings of this review support several public health policy priorities. First, strengthening AMR surveillance systems specific to DFIs should be a priority for health ministries and regional organizations. The Pan American Health Organization′s ReLAVRA+ network provides an existing framework that could be expanded to include DFI‐specific surveillance with standardized data collection, microbiological methods, and reporting formats [49]. Countries without existing microbiology capacity for DFI surveillance should receive technical and financial support to develop this capacity.

Second, national essential medicines list and treatment guidelines for DFIs should be updated to reflect current resistance patterns [50]. The high resistance to commonly used antibiotics documented in this review suggests that many existing guidelines may be outdated and could contribute to treatment failure and further resistance selection. Guidelines should be evidence‐based, regularly updated, and tailored to local resistance data where available.

Third, antimicrobial stewardship should be mandated or incentivized in hospitals and clinics caring for DFI patients. Regulatory requirements for stewardship programs, inclusion of stewardship metrics in hospital accreditation standards, and reimbursement models that support stewardship activities could accelerate implementation. The World Health Organization′s guidance on antimicrobial stewardship provides a framework adaptable to LAC settings.

Fourth, diabetes prevention and management programs should be strengthened as an indirect strategy to reduce the burden of DFIs and associated antibiotic use. The strong association between diabetes control and foot complication risk suggests that investments in diabetes care could reduce the population at risk for infected foot ulcers and consequently reduce antibiotic exposure and resistance selection pressure.

### 4.10. Implications for Future Research

This systematic review identifies numerous priorities for future research on AMR in DFIs in Latin America and the Caribbean. The most urgent priority is conducting primary research in the 18 countries with no eligible studies identified. These studies should employ standardized microbiological methods, use internationally recognized breakpoints, and report resistance data in a format that enables future meta‐analyses and cross‐country comparisons. Multicenter studies within countries and across the region would provide more representative data than single‐center studies and enable examination of intracountry variation.

Prospective cohort studies with adequate sample size and follow‐up duration are needed to better characterize the association between AMR and clinical outcomes. Such studies should collect detailed data on potential confounders including infection severity, comorbid conditions, prior antibiotic exposure, and surgical interventions, and should use standardized definitions for outcomes including amputation (minor vs. major), mortality, and treatment failure. Adjusted effect estimates from multivariable models would provide more reliable evidence than unadjusted comparisons.

Molecular epidemiology studies are needed to characterize the genetic basis of resistance in the region and to track the dissemination of resistance clones and plasmids. The detection of *mcr-1* in Brazil [17] and the high prevalence of integrons in Venezuelan *P. aeruginosa* [44] suggest that molecular surveillance could provide early warning of emerging resistance mechanisms and inform infection control responses. Regional networks for molecular surveillance could be established in collaboration with existing initiatives.

Interventional studies are needed to evaluate the effectiveness of antimicrobial stewardship programs, infection control interventions, and treatment algorithms tailored to the LAC context. Pragmatic trials of stewardship interventions embedded in routine clinical care could provide actionable evidence while building research capacity.

Health economic studies are needed to quantify the economic burden of AMR in DFIs and to evaluate the cost‐effectiveness of interventions. The Mexican study demonstrated substantial potential cost savings from early microbiological cultures [28], but similar analyses are lacking for other countries and for other interventions.

### 4.11. Evidence Strengths and Certainty

Based on GRADE assessment, the certainty of evidence for the primary outcomes was rated as moderate for MRSA prevalence (19 studies) and low for MDR and CR (11 and 9 studies, respectively). The moderate certainty for MRSA reflects a large number of studies with consistent direction of effect, downgraded for serious inconsistency (high heterogeneity). Low certainty for MDR and CR reflects limited study numbers and imprecision. Evidence for resistance‐outcome associations was rated as very low because of serious risk of bias, inconsistency, and imprecision. These certainty ratings should inform interpretation: Findings with moderate certainty (MRSA prevalence) provide reasonably reliable estimates; findings with low or very low certainty (MDR, carbapenem, outcome associations) should be interpreted with greater caution and considered hypothesis‐generating.

### 4.12. Strengths and Limitations of This Review

This systematic review and meta‐analysis has several strengths. The comprehensive search strategy across multiple databases and grey literature sources minimized the risk of missing relevant studies. The inclusion of studies in any language enabled identification of research not indexed in major databases, including thesis work and regional journals. The 31 included studies provided a substantial evidence base for meta‐analysis of key outcomes, with up to 19 studies contributing to MRSA estimates. The use of random‐effects models accounted for anticipated heterogeneity, and the exploration of heterogeneity through subgroup analyses provided insights into sources of variation.

However, several limitations must be considered. First, the substantial heterogeneity observed across studies for most outcomes limits the precision of pooled estimates and suggests that pooled results should be interpreted as summary measures that may not apply to specific localities or time periods. The high *I* ^2^ values for MRSA and MDR meta‐analyses indicate that variation between studies exceeds that expected by chance alone, and subgroup analyses did not fully explain this variation.

Second, the quality of included studies was variable, with many studies having methodological limitations including retrospective design, single‐center setting, small sample sizes, and incomplete reporting of methods and results. The lack of standardized breakpoint reporting in most studies raises questions about the comparability of resistance definitions across studies and time periods. Although sensitivity analyses excluding lower quality studies did not materially change results, residual confounding and bias may affect the validity of findings.

Third, publication bias may have influenced results, particularly for MRSA, where funnel plot asymmetry and a significant Egger′s test (*p* = 0.002) suggested possible under‐reporting of studies with low resistance prevalence. If studies finding low resistance rates are less likely to be published, pooled estimates may overestimate true prevalence. This possibility should be considered when interpreting the MRSA estimates. However, the inclusion of grey literature and thesis work may have partially mitigated this bias.

Fourth, the generalizability of findings is limited by the geographic concentration of research in a few countries. The 18 countries with no eligible studies represent a substantial proportion of the region, and resistance patterns in these settings may differ from those in studied countries. The findings of this review should therefore be considered most applicable to the countries and institutions where research has been conducted, with caution required when extrapolating to other settings.

Fifth, the limited availability of clinical outcome data restricted the ability to examine resistance–outcome associations meta‐analytically. Only four studies examined these associations, and only one provided adjusted effect estimates. The relationship between resistance and patient‐important outcomes, therefore, remains incompletely characterized in the LAC context.

Sixth, the absence of patient‐level data precluded more nuanced analyses of resistance patterns by clinical characteristics such as infection severity, prior antibiotic exposure, and healthcare setting. Meta‐analyses of aggregate data cannot examine these individual‐level associations.

## 5. Conclusions

This systematic review and meta‐analysis documents that AMR in DFIs is highly prevalent and increasing in Latin America and the Caribbean, with approximately one‐third of *S. aureus* isolates methicillin‐resistant, one‐third of Enterobacterales producing ESBLs, and more than half of patients harboring MDR organisms. ESBL rates vary substantially across settings, and CR, while lower, remains a significant concern. The clinical consequences are substantial, with exploratory evidence suggesting MDR infections are associated with increased risks of amputation and prolonged hospitalization. Geographic heterogeneity is marked, with resistance rates varying substantially across countries and institutions, and critical knowledge gaps exist in 18 countries where no primary research has been conducted. These findings support urgent implementation of antimicrobial stewardship programs, infection prevention and control measures, and strengthened surveillance systems specifically targeting DFI care in the region. Without concerted action at clinical, institutional, and policy levels, the substantial gains made in diabetes management risk being undermined by the growing threat of untreatable infections.

## Author Contributions

D.C.H.: conceptualization, methodology, data curation, formal analysis, visualizations, supervision, writing—original draft, editing, project administration. J.H.C.: conceptualization, methodology, data curation, formal analysis, writing—original draft. G.M.S.: conceptualization, methodology, data curation, formal analysis, writing—original draft. C.E.I.: methodology, validation, writing review and final editing. I.N.: methodology, validation, writing review and final editing. C.N.: supervision, formal analysis, validation, writing—review and final editing. O.E.O.: supervision, formal analysis, validation, writing—review and final editing.

## Funding

No funding was received for this manuscript.

## Disclosure

All the authors read and approved the final manuscript.

## Ethics Statement

The authors have nothing to report.

## Consent

The authors have nothing to report.

## Conflicts of Interest

The authors declare no conflicts of interest.

## Supporting information


**Supporting Information** Additional supporting information can be found online in the Supporting Information section. Table S1: PRISMA 2020 main checklist. The table documents compliance with reporting guidelines for systematic reviews and meta‐analyses. Table S2: Detailed methodological characteristics of included studies (*n* = 31). The table includes study design, study period, setting, healthcare level, sampling method, and risk of bias scores. Table S3: Detailed participant characteristics of included studies (*n* = 31). The table contains patient age, sex, wound classification, infection severity, and number of isolates. Table S4: Detailed microbiological methods of included study (*N* = 31). Culture method, bacterial identification method, antimicrobial susceptibility testing method, and breakpoint standards used. Table S5: Country‐specific pooled methicillin‐resistant *Staphylococcus aureus* Prevalence Estimates (19 Studies) Table S6: Extended‐spectrum *β*‐lactamase (ESBL) prevalence reported in included studies. Table S7: Pooled resistance rates to specific antibiotic classes. Meta‐analyses of 3–17 studies per antibiotic. Table S8: Risk of bias assessments for included studies (*n* = 31). The Newcastle–Ottawa Scale for cohort studies and the Joanna Briggs Institute checklist for cross‐sectional studies. Table S9: GRADE assessment of certainty of evidence (31 studies). Figure S1: Funnel plots for assessment of publication bias. (A) MRSA prevalence (19 studies) and (B) multidrug‐resistant (MDR) prevalence (11 studies).

## Data Availability

All data supporting the findings of this study are included within the manuscript and its supporting materials.
