# Supplementary material for: Antimicrobial Resistance in Diabetic Foot Infections in Latin America and the Caribbean: A Systematic Review and Meta‐Analysis (1980–2025)
Source: Int J Microbiol. 2026 Aug 25;2026:5244269. doi: 10.1155/ijm/5244269 (PMC13507709; doi:10.1155/ijm/5244269)
Supplement: Supplementary file 1 [file IJM-2026-5244269-s001.docx]

# Antimicrobial Resistance in Diabetic Foot Infections in Latin America and the Caribbean: A Systematic Review and Meta-Analysis (1980–2025)

Danladi C. Husaini¹^,8^*, Joel H. Chiroma², Garba M. Sani³, Collins E. Ihebie⁴, Innocent Nwachukwu⁵^,8^, Chukwuemeka Nwokocha^6^, and Orish E. Orisakwe^7^

**Supplementary Material**

**Supplementary Table S1: PRISMA 2020 Main Checklist**

| Section and Topic | Item # | Checklist Item | Location where item is reported |
| --- | --- | --- | --- |
| TITLE |  |  |  |
| Title | 1 | Identify the report as a systematic review. | Title page |
| ABSTRACT |  |  |  |
| Abstract | 2 | See the PRISMA 2020 for Abstracts checklist. | Abstract |
| INTRODUCTION |  |  |  |
| Rationale | 3 | Describe the rationale for the review in the context of existing knowledge. | Introduction, paragraphs 1–4 |
| Objectives | 4 | Provide an explicit statement of the objective(s) or question(s) the review addresses. | Introduction, paragraph 6 |
| METHODS |  |  |  |
| Eligibility criteria | 5 | Specify the inclusion and exclusion criteria for the review and how studies were grouped for the syntheses. | Methods, Eligibility Criteria, Table 2 |
| Information sources | 6 | Specify all databases, registers, websites, organizations, reference lists and other sources searched or consulted to identify studies. Specify the date when each source was last searched or consulted. | Methods, Search Strategy |
| Search strategy | 7 | Present the full search strategies for all databases, registers and websites, including any filters and limits used. | Methods, Search Strategy; Supplementary Appendix 1 |
| Selection process | 8 | Specify the methods used to decide whether a study met the inclusion criteria of the review, including how many reviewers screened each record and each report retrieved, whether they worked independently, and if applicable, details of automation tools used. | Methods, Study Selection Process |
| Data collection process | 9 | Specify the methods used to collect data from reports, including how many reviewers collected data from each report, whether they worked independently, any processes for obtaining or confirming data from study investigators, and if applicable, details of automation tools used. | Methods, Data Extraction |
| Data items | 10a | List and define all outcomes for which data were sought. Specify whether all results that were compatible with each outcome domain in each study were sought (e.g. for all measures, time points, analyses), and if not, the methods used to decide which results to collect. | Methods, Data Extraction; PICO Table 1 |
|  | 10b | List and define all other variables for which data were sought (e.g. participant and intervention characteristics, funding sources). Describe any assumptions made about any missing or unclear information. | Methods, Data Extraction |
| Study risk of bias assessment | 11 | Specify the methods used to assess risk of bias in the included studies, including details of the tool(s) used, how many reviewers assessed each study and whether they worked independently, and if applicable, details of automation tools used. | Methods, Risk of Bias Assessment |
| Effect measures | 12 | Specify for each outcome the effect measure(s) (e.g. risk ratio, mean difference) used in the synthesis or presentation of results. | Methods, Data Synthesis and Statistical Analysis |
| Synthesis methods | 13a | Describe the processes used to decide which studies were eligible for each synthesis (e.g. tabulating the study intervention characteristics and comparing against the planned groups for each synthesis (item #5). | Methods, Data Synthesis and Statistical Analysis |
|  | 13b | Describe any methods required to prepare the data for presentation or synthesis, such as handling of missing summary statistics, or data conversions. | Methods, Data Synthesis and Statistical Analysis |
|  | 13c | Describe any methods used to tabulate or visually display results of individual studies and syntheses. | Methods, Data Synthesis and Statistical Analysis |
|  | 13d | Describe any methods used to synthesize results and provide a rationale for the choice(s). If meta-analysis was performed, describe the model(s), method(s) to identify the presence and extent of statistical heterogeneity, and software package(s) used. | Methods, Data Synthesis and Statistical Analysis |
|  | 13e | Describe any methods used to explore possible causes of heterogeneity among study results (e.g. subgroup analysis, meta-regression). | Methods, Data Synthesis and Statistical Analysis |
|  | 13f | Describe any sensitivity analyses conducted to assess robustness of the synthesized results. | Methods, Data Synthesis and Statistical Analysis |
| Reporting bias assessment | 14 | Describe any methods used to assess risk of bias due to missing results in a synthesis (arising from reporting biases). | Methods, Data Synthesis and Statistical Analysis |
| Certainty assessment | 15 | Describe any methods used to assess certainty (or confidence) in the body of evidence for an outcome. | Methods, Assessment of Certainty of Evidence |
| RESULTS |  |  |  |
| Study selection | 16a | Describe the results of the search and selection process, from the number of records identified in the search to the number of studies included in the review, ideally using a flow diagram. | Results, Study Selection; Figure 1 |
|  | 16b | Cite studies that might appear to meet the inclusion criteria, but which were excluded, and explain why they were excluded. | Supplementary Appendix 2 |
| Study characteristics | 17 | Cite each included study and present its characteristics. | Results, Study Characteristics; Tables 3–4; Supplementary Table S2 |
| Risk of bias in studies | 18 | Present assessments of risk of bias for each included study. | Supplementary Table S8 |
| Results of individual studies | 19 | For all outcomes, present, for each study: (a) summary statistics for each group (where appropriate) and (b) an effect estimate and its precision (e.g. confidence/credible interval), ideally using structured tables or plots. | Results, Tables 3–5; Figures 2–6; Supplementary Tables S5–S7 |
| Results of syntheses | 20a | For each synthesis, briefly summarize the characteristics and risk of bias among contributing studies. | Results, Antimicrobial Resistance Outcomes; Risk of Bias Assessment |
|  | 20b | Present results of all statistical syntheses conducted. If meta-analysis was done, present for each the summary estimate and its precision (e.g. confidence/credible interval) and measures of statistical heterogeneity. | Results, Antimicrobial Resistance Outcomes; Figures 3,5,6 |
|  | 20c | Present results of all investigations of possible causes of heterogeneity among study results. | Results, Subgroup analyses; Meta-regression; Figures 4 |
|  | 20d | Present results of all sensitivity analyses conducted to assess the robustness of the synthesized results. | Results, Sensitivity Analyses |
| Reporting biases | 21 | Present assessments of risk of bias due to missing results (arising from reporting biases) for each synthesis assessed. | Results, Publication Bias; Supplementary Figure S1 |
| Certainty of evidence | 22 | Present assessments of certainty (or confidence) in the body of evidence for each outcome assessed. | Results, Certainty of Evidence; Supplementary Table S9 |
| DISCUSSION |  |  |  |
| Discussion | 23a | Provide a general interpretation of the results in the context of other evidence. | Discussion, Comparison with Global Evidence |
|  | 23b | Discuss any limitations of the evidence included in the review. | Discussion, Strengths and Limitations |
|  | 23c | Discuss any limitations of the review processes used. | Discussion, Strengths and Limitations |
|  | 23d | Discuss implications of the results for practice, policy, and future research. | Discussion, Clinical Implications; Policy Implications; Future Research |
| OTHER INFORMATION |  |  |  |
| Registration and protocol | 24a | Provide registration information for the review, including register name and registration number, or state that the review was not registered. | Methods, paragraph 1 |
|  | 24b | Indicate where the review protocol can be accessed, or state that a protocol was not prepared. | Methods, paragraph 1 |
|  | 24c | Describe and explain any amendments to information provided at registration or in the protocol. | Not applicable |
| Support | 25 | Describe sources of financial or non-financial support for the review, and the role of the funders or sponsors in the review. | Abstract, Funding; Acknowledgements |
| Competing interests | 26 | Declare any competing interests of review authors. | Conflicts of Interest statement |
| Availability of data, code and other materials | 27 | Report which of the following are publicly available and where they can be found: template data collection forms; data extracted from included studies; data used for all analyses; analytic code; any other materials used in the review. | Available from corresponding author upon reasonable request |

**Supplementary Table S2: Detailed Methodological Characteristics of Included Studies (n = 31)**

| ID | First Author (Year) | Study Design | Study Period | Setting | City, Country | Healthcare Level | Sampling Method | Risk of Bias Score |
| --- | --- | --- | --- | --- | --- | --- | --- | --- |
| 1 | Cervantes-García et al. 2017 | Longitudinal descriptive | Jul 2012–Aug 2015 | Hospital General de México | Mexico City, Mexico | Tertiary | Consecutive patients | NOS: 5/9 |
| 2 | Chin-Harty et al. 2021 | Retrospective | Jan 2003–Dec 2008 | University Hospital of the West Indies | Kingston, Jamaica | Tertiary | Consecutive patients | NOS: 7/9 |
| 3 | Islam et al. 2013a | Prospective | Jul 2011–Jun 2012 | Tertiary hospitals | Trinidad and Tobago | Tertiary | Consecutive patients | NOS: 8/9 |
| 4 | Islam et al. 2013b | Prospective with retrospective chart review | Jul 2011–Jun 2012 | Tertiary care facility | Trinidad and Tobago | Tertiary | Consecutive patients | NOS: 8/9 |
| 5 | Víquez-Molina et al. 2018 | Cross-sectional | Jun 2014–May 2016 | San Juan de Dios Hospital | San José, Costa Rica | Tertiary | Consecutive patients | JBI: 7/8 |
| 6 | Carro et al. 2022 | Multicenter cross-sectional | Sep–Dec 2019 | 17 centers in 4 countries | Argentina, El Salvador, Chile, Guatemala | Mixed | Consecutive patients | JBI: 8/8 |
| 7 | Carvalho et al. 2004 | Prospective | Mar 2000–Nov 2001 | Federal University of Ceará | Fortaleza, Brazil | Tertiary | Consecutive patients | NOS: 7/9 |
| 8 | Cascante-Serrano et al. 2021 | Retrospective descriptive | 2018 | San Juan de Dios Hospital | San José, Costa Rica | Tertiary | Consecutive patients | JBI: 6/8 |
| 9 | Carro et al. 2019 | Descriptive observational | Mar–Sep 2018 | Hospital Nacional Posadas | Buenos Aires, Argentina | Tertiary | Consecutive patients | JBI: 7/8 |
| 10 | Sánchez-Sánchez et al. 2017 | Observational | Dec 2011–Apr 2016 | Angiology and Vascular Surgery Hospital | Reynosa, Mexico | Tertiary | Consecutive patients | JBI: 7/8 |
| 11 | Cervantes-García et al. 2015a | Prospective | Jul 2012–Dec 2013 | Hospital General de México | Mexico City, Mexico | Tertiary | Consecutive patients | JBI: 6/8 |
| 12 | Cervantes-García et al. 2015b | Cross-sectional | Jul 2012–Dec 2013 | Hospital General de México | Mexico City, Mexico | Tertiary | Consecutive patients | JBI: 6/8 |
| 13 | Zambelli et al. 2025 | Retrospective cohort | 2013–2022 | Private tertiary hospital | Belo Horizonte, Brazil | Tertiary | Consecutive patients | NOS: 6/9 |
| 14 | Copari-Vargas et al. 2025 | Retrospective | Jan 2014–Dec 2018 | Hospital de Clínicas | La Paz, Bolivia | Secondary | Consecutive patients | JBI: 7/8 |
| 15 | Falcón et al. 2006 | Descriptive | Jun 2000–May 2005 | Hospital Arzobispo Loayza | Lima, Peru | Tertiary | Consecutive patients | JBI: 5/8 |
| 16 | Fernandes et al. 2016 | Case report / molecular | Not reported | Private hospital | Northeastern Brazil | Tertiary | Single case | JBI Case Series: N/A |
| 17 | Rodulfo et al. 2019 | Longitudinal observational | 2009–2016 | HUAPA | Cumaná, Venezuela | Tertiary | Consecutive patients | NOS: 7/9 |
| 18 | Kurup et al. 2019 | Prospective cross-sectional | Nov 2016–Feb 2017 | Georgetown Public Hospital | Georgetown, Guyana | Tertiary | Consecutive patients | JBI: 6/8 |
| 19 | Leiva Jarquín 2022 | Retrospective cross-sectional (thesis) | Jul–Nov 2022 | HEODRA Hospital | León, Nicaragua | Tertiary | Consecutive patients | JBI: 6/8 |
| 20 | Carrión et al. 2017 | Retrospective descriptive | Jan 2012–Dec 2016 | Hospital Rawson | San Juan, Argentina | Tertiary | Consecutive patients | JBI: 5/8 |
| 21 | Sanchez et al. 2022 | Retrospective descriptive | Jan 2013–Jun 2019 | Samaritana University Hospital | Bogotá, Colombia | Tertiary | Consecutive patients | JBI: 7/8 |
| 22 | Motta et al. 2003 | Prospective | Mar 2000–Nov 2001 | Federal University of Ceará | Fortaleza, Brazil | Tertiary | Consecutive patients | NOS: 7/9 |
| 23 | Moya-Salazar et al. 2023 | Retrospective cross-sectional | 2019 | Hospital Maria Auxiliadora | Lima, Peru | Tertiary | Consecutive patients | NOS: 7/9 |
| 24 | Yovera-Aldana et al. 2024 | Retrospective cohort | 2017–2019 | Hospital Maria Auxiliadora | Lima, Peru | Tertiary | Consecutive patients | NOS: 8/9 |
| 25 | Dos Santos et al. 2024 | Cross-sectional | Dec 2014–Jan 2018 | Complexo Hospitalar Universitário | Salvador, Brazil | Tertiary | Consecutive patients | JBI: 7/8 |
| 26 | Palomo et al. 2022 | Retrospective | 2017–2019 | Hospital do Servidor Público Estadual | São Paulo, Brazil | Tertiary | Consecutive patients | NOS: 6/9 |
| 27 | Perim et al. 2015 | Prospective | Jan–Jun 2013 | Hospital Geral de Palmas | Palmas, Brazil | Tertiary | Consecutive patients | JBI: 7/8 |
| 28 | Rojas Tapia et al. 2023 | Cross-sectional | Sep 2021–May 2022 | Hospital Universitario del Caribe | Cartagena, Colombia | Tertiary | Consecutive patients | JBI: 7/8 |
| 29 | Nascimento et al. 2024 | Cross-sectional | 2017–2018 | Regional public hospital | Vitória da Conquista, Brazil | Secondary | Consecutive patients | JBI: 7/8 |
| 30 | Pontes et al. 2020 | Prospective cross-sectional | Mar–Aug 2018 | Hospital 28 de Agosto | Manaus, Brazil | Tertiary | Consecutive patients | JBI: 7/8 |
| 31 | Balderas-Peña et al. 2016 | Prospective longitudinal | Jan–Apr 2010 | IMSS second-level facility | Guadalajara, Mexico | Secondary | Consecutive patients | NOS: 6/9 |

*NOS = Newcastle-Ottawa Scale; JBI = Joanna Briggs Institute. Scores for case reports are indicated where applicable.*

**Supplementary Table S3: Detailed Participant Characteristics of Included Studies (n=31)**

| ID | First Author (Year) | Patients (n) | Isolates (n) | Mean/Median Age (years) | Male (%) | Inclusion Criteria | Wound Classification | Infection Severity |
| --- | --- | --- | --- | --- | --- | --- | --- | --- |
| 1 | Cervantes‑García et al. 2017 | 100 | 100 | 52 ± 10.2 (M), 50 ± 10.4 (F) | 60% | T2DM, infected DFU | Wagner 2–5 | PEDIS 2–4 |
| 2 | Chin‑Harty et al. 2021 | 102 | 102 | 60.2 ± 14.5 | 46% | DFI, admitted | Wagner 2–5 | Sepsis 47.1% |
| 3 | Islam et al. 2013a | 446 | Not reported | 56.9 ± 12.4 | 54.0% | DFI admitted | Not reported | Not reported |
| 4 | Islam et al. 2013b | 139 | 221 | 56.9 ± 12.4 | Not reported | Moderate‑severe DFI | Not reported | Moderate (26%), severe (74%) |
| 5 | Víquez‑Molina et al. 2018 | 58 | 58 (*S. aureus*) | 58.2 ± 12.4 | Not reported | Monomicrobial S. aureus DFI | Not reported | Moderate 37.9%, severe 62.1% |
| 6 | Carro et al. 2022 | 307 positive samples | 418 | 60 (median) | 70% | DFI, first presentation | Not reported | Not reported |
| 7 | Carvalho et al. 2004 | 141 | 298 | Not reported | 54.6% | Community‑acquired DFU | Wagner I–II | Mild to moderate |
| 8 | Cascante‑Serrano et al. 2021 | 48 | 73 | 61–70 (40%) | 69% | DF osteomyelitis | Not reported | Osteomyelitis |
| 9 | Carro et al. 2019 | 267 | 68 (positive) | 60.2 ± 11.7 | 63.7% | DFI outpatients | Not reported | Not reported |
| 10 | Sánchez‑Sánchez et al. 2017 | 215 | 246 | 58.9 ± 11.5 | 60.4% | DFU, culture taken | Wagner 2–3 (86.5%) | Not reported |
| 11 | Cervantes‑García et al. 2015a | 120 | 120 | Not reported | Not reported | T2DM, infected DFU | Wagner 3–5 | Not reported |
| 12 | Cervantes‑García et al. 2015b | 100 | 100 | 51.6 (mean) | Not reported | T2DM, infected DFU | Wagner 2–5 | Not reported |
| 13 | Zambelli et al. 2025 | 337 | 507 | 66.4 ± 13.8 | 69.1% | DFI with positive culture | Not reported | Not reported |
| 14 | Copari‑Vargas et al. 2025 | 28 | 28 | 59.4 ± 2.3 | 35.7% | DFI, complete data | Wagner 2–3 (57.2%) | Sepsis 7.1% |
| 15 | Falcón et al. 2006 | 95 | 132 | 61.7 ± 11.6 | 34% | DFI with positive culture | Wagner 3 (51.4%) | Not reported |
| 16 | Fernandes et al. 2016 | 1 | 1 | Late 60s | Male | Single DFI case | Not reported | Not reported |
| 17 | Rodulfo et al. 2019 | 41 (DFI subset) | 41 (*P. aeruginosa*) | Not reported | Not reported | DFI among hospitalized | Not reported | Not reported |
| 18 | Kurup et al. 2019 | 109 (DF) | 254 total | 60.0 ± 17.0 (MDR) | 51.6% (MDR) | DFU, outpatient clinic | Not reported | Mild–severe |
| 19 | Leiva Jarquín 2022 | 66 | 66 | >50 (80.3%) | 68.2% | DFI hospitalized | WIfI | WIfI II (54.5%), III (40.9%) |
| 20 | Carrión et al. 2017 | 143 samples | 143 | Not reported | Not reported | DFI, outpatients and hospitalized | Not reported | Not reported |
| 21 | Sanchez et al. 2022 | 186 | 289 | Not reported | Not reported | DFI, ulcer culture | Not reported | Not reported |
| 22 | Motta et al. 2003 | 138 | 156 | Not reported | Not reported | Community‑acquired DFU | Not reported | Not reported |
| 23 | Moya‑Salazar et al. 2023 | 181 | 181 | 61–70 (35.9%) | 70.7% | T2DM, DFI, positive culture | Not reported | Not reported |
| 24 | Yovera‑Aldana et al. 2024 | 192 | 236 | 59.9 ± 12.9 | 74.0% | DFI treated at DFU | Wagner IV (50.5%) | IDSA moderate‑severe 88% |
| 25 | Dos Santos et al. 2024 | 111 | 185 | 63.6 ± 12.8 | 48% | Moderate‑severe lower extremity wounds | Wagner ≥3, WIfI 2–3 | IDSA moderate‑severe 82% |
| 26 | Palomo et al. 2022 | 320 | 276 | 71 (mean) | 69.7% | DFI requiring hospitalization | IWGDF III (52.8%) | Severe (52.8%) |
| 27 | Perim et al. 2015 | 41 | 89 | 65.8 ± 13.8 | 46% | DFU requiring debridement | Not reported | Not reported |
| 28 | Rojas Tapia et al. 2023 | 149 | 149 | 61 (median) | 61.8% | DFI, hospitalized | Wagner IV (30.2%) | Moderate‑severe 67.8% |
| 29 | Nascimento et al. 2024 | 34 | 17 (*S. aureu*s) | 60 (mean) | 70.6% | DFU with amputation indication | Wagner IV (52.9%), V (26.5%) | Severe |
| 30 | Pontes et al. 2020 | 105 | 105 | 57.8 ± 11.6 | 60.0% | DFI requiring surgery | Wagner 2 (54.3%), 3 (34.3%) | PEDIS 3 (79%) |
| 31 | Balderas‑Peña et al. 2016 | 67 | 67 | 56.1 ± 12.3 | 81.2% | DFU, infected | Wagner 3 (43.5%), 4 (31.9%) | Not reported |

DF = diabetic foot; DFI = diabetic foot infection; DFU = diabetic foot ulcer; T2DM = type 2 diabetes mellitus; MDR = multidrug-resistant.

**Supplementary Table S4: Detailed Microbiological Methods of Included Studies (n=31)**

| ID | First Author (Year) | Culture Method | Identification Method | AST Method | Breakpoints Used |
| --- | --- | --- | --- | --- | --- |
| 1 | Cervantes‑García et al. 2017 | Swab after debridement | Conventional | Kirby‑Bauer | CLSI |
| 2 | Chin‑Harty et al. 2021 | Not specified | Conventional | Not specified | Not reported |
| 3 | Islam et al. 2013a | Not specified | Not specified | Not specified | Not reported |
| 4 | Islam et al. 2013b | Aspiration, debridement, swab | Not specified | Kirby‑Bauer | Not reported |
| 5 | Víquez‑Molina et al. 2018 | Soft tissue/bone biopsy | Conventional + PCR | Kirby‑Bauer | CLSI |
| 6 | Carro et al. 2022 | Soft tissue/bone biopsy | Conventional + automated | Kirby‑Bauer, MIC | CLSI (inferred) |
| 7 | Carvalho et al. 2004 | Selective media | Conventional + automated | Kirby‑Bauer, MIC | NCCLS (CLSI) |
| 8 | Cascante‑Serrano et al. 2021 | Bone biopsy | Conventional | Kirby‑Bauer | Not reported |
| 9 | Carro et al. 2019 | Fine‑needle puncture | Not specified | Vitek 2 | Not reported |
| 10 | Sánchez‑Sánchez et al. 2017 | Tissue biopsy or swab | Conventional | Kirby‑Bauer | CLSI |
| 11 | Cervantes‑García et al. 2015a | Swab after debridement | Conventional + SCV‑specific | Kirby‑Bauer | CLSI |
| 12 | Cervantes‑García et al. 2015b | Swab after debridement | Conventional | Kirby‑Bauer | CLSI |
| 13 | Zambelli et al. 2025 | Tissue (intraoperative) | Not reported | Not reported | Not reported |
| 14 | Copari‑Vargas et al. 2025 | Not specified | Not specified | Not specified | Not reported |
| 15 | Falcón et al. 2006 | Debrided ulcer base | Conventional | Kirby‑Bauer | Not reported |
| 16 | Fernandes et al. 2016 | Wound culture | WGS | MIC | CLSI/EUCAST |
| 17 | Rodulfo et al. 2019 | Not specified | Conventional + PCR | Kirby‑Bauer | Not reported |
| 18 | Kurup et al. 2019 | Swab, curette, pus | Conventional | Kirby‑Bauer | CLSI |
| 19 | Leiva Jarquín 2022 | Wound culture | Not specified | Not specified | Not reported |
| 20 | Carrión et al. 2017 | Not specified | Not specified | Not specified | Not reported |
| 21 | Sanchez et al. 2022 | Not specified | Not specified | Not specified | Not reported |
| 22 | Motta et al. 2003 | Selective media | Conventional + automated | Kirby‑Bauer, MIC | NCCLS (CLSI) |
| 23 | Moya‑Salazar et al. 2023 | Wound sample | Vitek 2 | Vitek 2 | CLSI M100 |
| 24 | Yovera‑Aldana et al. 2024 | Tissue biopsy | Not specified | Not specified | Not reported |
| 25 | Dos Santos et al. 2024 | Tissue (intraoperative) | Vitek 2 | Vitek 2 | CLSI |
| 26 | Palomo et al. 2022 | Tissue (intraoperative) | Phoenix automated | Phoenix automated | CLSI |
| 27 | Perim et al. 2015 | Swab after debridement | Conventional | Kirby‑Bauer, MIC (vancomycin, polymyxin B) | CLSI |
| 28 | Rojas Tapia et al. 2023 | Not specified | Not specified | Not specified | Not reported |
| 29 | Nascimento et al. 2024 | Infected tissue | Conventional + PCR | Disk diffusion, MIC | CLSI |
| 30 | Pontes et al. 2020 | Tissue biopsy (intraoperative) | Conventional | Kirby‑Bauer, MIC | CLSI |
| 31 | Balderas‑Peña et al. 2016 | Not specified | Not specified | Not specified | Not reported |

*WGS = whole‑genome sequencing; PCR = polymerase chain reaction; SCV = small colony variant; NCCLS = National Committee for Clinical Laboratory Standards (now CLSI).*

## Supplementary Table S5: Country-Specific Pooled Methicillin-Resistant Staphylococcus aureus Prevalence Estimates (19 Studies)

| Country | Number of Studies | Pooled MRSA Prevalence (%) | 95% Confidence Interval | I² (%) |
| --- | --- | --- | --- | --- |
| Brazil | 7 | 30.2 | 24.1–36.7 | 88.2 |
| Mexico | 3 | 56.2 | 43.3–68.5 | 92.0 |
| Peru | 2 | 58.7 | 48.8–68.1 | 63.4 |
| Costa Rica | 2 | 65.2 | 57.6–72.4 | 0.0 |
| Argentina | 1 | 14.8 | – | – |
| Bolivia | 1 | 20.0 | – | – |
| Guyana | 1 | 60.6 | – | – |
| Jamaica | 1 | 0.0 | – | – |
| Trinidad and Tobago | 1 | 15.2 | – | – |
| **Overall Pooled** | **19** | **37.8** | **31.8–44.1** | **94.1** |

Note: Pooled estimates for countries with two or more studies were calculated using random-effects meta-analysis. Single-study estimates are presented without confidence intervals.

## Supplementary Table S6: Extended-Spectrum β‑Lactamase (ESBL) Prevalence in Included Studies

| Study (First Author, Year) | Country | ESBL Events | Total Enterobacterales | ESBL Prevalence (%) |
| --- | --- | --- | --- | --- |
| Motta et al. 2003 | Brazil | 10 | 156 | 6.4 |
| Islam et al. 2013b | Trinidad and Tobago | 25 | 143* | 17.5 |
| Zambelli et al. 2025 | Brazil | 32 | 208 | 15.4 |
| Dos Santos et al. 2024 | Brazil | 45 | 133 | 33.8 |
| Pontes et al. 2020 | Brazil | 8 | 57† | 14.0 |
| Carro et al. 2022 | Multicenter (LAC) | – | – | 34.2‡ |

*ESBL events are among Gram-negative isolates (total 143).
†ESBL events are among Gram-negative isolates (total 57).
‡Percentage reported; exact denominator not available.

## Supplementary Table S7: Pooled Resistance Rates to Specific Antibiotic Classes

| Antibiotic Class / Agent | Organism Group | Number of Studies | Pooled Resistance (%) | 95% Confidence Interval | I² (%) |
| --- | --- | --- | --- | --- | --- |
| Ampicillin | Gram-negative bacilli | 9 | 81.9 | 77.4–86.0 | 86.4 |
| Trimethoprim-sulfamethoxazole | All isolates | 14 | 60.1 | 55.6–64.5 | 89.1 |
| Ciprofloxacin | All isolates | 17 | 46.3 | 41.8–50.9 | 91.7 |
| Gentamicin | S. aureus | 7 | 29.1 | 20.2–39.0 | 88.3 |
| Erythromycin | S. aureus | 5 | 42.7 | 32.1–53.8 | 90.2 |
| Clindamycin | S. aureus | 4 | 32.4 | 20.8–45.3 | 89.5 |
| Tetracycline | S. aureus | 3 | 30.1 | 22.4–38.5 | 76.8 |
| Vancomycin | S. aureus | 8 | 0.0 | – | 0.0 |
| Linezolid | S. aureus | 4 | 0.0 | – | 0.0 |

## Supplementary Table S8: Risk of Bias Assessments for Included Studies (n = 31)

### **Newcastle-Ottawa Scale (NOS) for Cohort Studies**

| ID | Author (Year) | Selection (max 4) | Comparability (max 2) | Outcome (max 3) | Total Score (max 9) |
| --- | --- | --- | --- | --- | --- |
| 1 | Cervantes-García et al. 2017 | 2 | 1 | 2 | 5 |
| 2 | Chin-Harty et al. 2021 | 3 | 2 | 2 | 7 |
| 3 | Islam et al. 2013a | 4 | 2 | 2 | 8 |
| 4 | Islam et al. 2013b | 4 | 2 | 2 | 8 |
| 5 | Carvalho et al. 2004 | 3 | 2 | 2 | 7 |
| 6 | Zambelli et al. 2025 | 3 | 1 | 2 | 6 |
| 7 | Rodulfo et al. 2019 | 3 | 2 | 2 | 7 |
| 8 | Motta et al. 2003 | 3 | 2 | 2 | 7 |
| 9 | Moya-Salazar et al. 2023 | 3 | 2 | 2 | 7 |
| 10 | Yovera-Aldana et al. 2024 | 4 | 2 | 2 | 8 |
| 11 | Palomo et al. 2022 | 3 | 1 | 2 | 6 |
| 12 | Balderas-Peña et al. 2016 | 3 | 1 | 2 | 6 |

### **Joanna Briggs Institute (JBI) Checklist for Cross-Sectional Studies**

| ID | Author (Year) | Q1 | Q2 | Q3 | Q4 | Q5 | Q6 | Q7 | Q8 | Total Score (max 8) |
| --- | --- | --- | --- | --- | --- | --- | --- | --- | --- | --- |
| 13 | Víquez-Molina et al. 2018 | Y | Y | Y | Y | Y | Y | Y | N | 7 |
| 14 | Carro et al. 2022 | Y | Y | Y | Y | Y | Y | Y | Y | 8 |
| 15 | Cascante-Serrano et al. 2021 | Y | Y | Y | N | Y | Y | Y | N | 6 |
| 16 | Carro et al. 2019 | Y | Y | Y | Y | Y | Y | Y | N | 7 |
| 17 | Sánchez-Sánchez et al. 2017 | Y | Y | Y | Y | Y | Y | Y | N | 7 |
| 18 | Cervantes-García et al. 2015a | Y | Y | Y | N | Y | Y | Y | N | 6 |
| 19 | Cervantes-García et al. 2015b | Y | Y | Y | N | Y | Y | Y | N | 6 |
| 20 | Copari-Vargas et al. 2025 | Y | Y | Y | Y | Y | Y | Y | N | 7 |
| 21 | Falcón et al. 2006 | Y | N | Y | N | Y | Y | Y | N | 5 |
| 22 | Kurup et al. 2019 | Y | Y | Y | N | Y | Y | Y | N | 6 |
| 23 | Leiva Jarquín 2022 | Y | Y | Y | N | Y | Y | Y | N | 6 |
| 24 | Carrión et al. 2017 | Y | N | Y | N | Y | N | Y | N | 5 |
| 25 | Sanchez et al. 2022 | Y | Y | Y | Y | Y | Y | Y | N | 7 |
| 26 | Dos Santos et al. 2024 | Y | Y | Y | Y | Y | Y | Y | N | 7 |
| 27 | Perim et al. 2015 | Y | Y | Y | Y | Y | Y | Y | N | 7 |
| 28 | Rojas Tapia et al. 2023 | Y | Y | Y | Y | Y | Y | Y | N | 7 |
| 29 | Nascimento et al. 2024 | Y | Y | Y | Y | Y | Y | Y | N | 7 |
| 30 | Pontes et al. 2020 | Y | Y | Y | Y | Y | Y | Y | N | 7 |
| 31 | Fernandes et al. 2016* | – | – | – | – | – | – | – | – | N/A |

*Case report; no risk of bias assessment performed using JBI (assessed as low methodological quality but included for molecular resistance data only).

**JBI questions:** Q1: clear inclusion criteria; Q2: subjects and setting described; Q3: valid exposure measurement; Q4: objective, standard criteria for condition; Q5: confounding identified; Q6: strategies to deal with confounding stated; Q7: valid outcome measurement; Q8: appropriate statistical analysis. Y = Yes; N = No or Unclear.

## Supplementary Table S9: GRADE Assessment of Certainty of Evidence (31 Studies)

| Outcome | Number of Studies | Study Design | Risk of Bias | Inconsistency | Indirectness | Imprecision | Publication Bias | Overall Certainty | Importance |
| --- | --- | --- | --- | --- | --- | --- | --- | --- | --- |
| MRSA prevalence | 19 | Observational | Not serious | Serious¹ | Not serious | Not serious | Suspected² | ⨁⨁⨁◯ MODERATE | Critical |
| MDR prevalence | 11 | Observational | Not serious | Serious¹ | Not serious | Serious³ | Not detected | ⨁⨁◯◯ LOW | Critical |
| Carbapenem resistance | 9 | Observational | Not serious | Serious¹ | Not serious | Serious³ | Undetected | ⨁⨁◯◯ LOW | Important |
| Ampicillin resistance | 9 | Observational | Not serious | Serious¹ | Not serious | Not serious | Undetected | ⨁⨁⨁◯ MODERATE | Important |
| TMP-SMX resistance | 14 | Observational | Not serious | Serious¹ | Not serious | Not serious | Undetected | ⨁⨁⨁◯ MODERATE | Important |
| Ciprofloxacin resistance | 17 | Observational | Not serious | Serious¹ | Not serious | Not serious | Undetected | ⨁⨁⨁◯ MODERATE | Important |
| Resistance-amputation association | 1 | Observational | Serious⁴ | Not applicable | Not serious | Serious⁵ | Undetected | ⨁◯◯◯ VERY LOW | Critical |
| Resistance-mortality association | 1 | Observational | Serious⁴ | Not applicable | Not serious | Very serious⁶ | Undetected | ⨁◯◯◯ VERY LOW | Critical |

**Footnotes:**
¹ Serious inconsistency: I² > 75% indicating substantial heterogeneity not fully explained by subgroup analyses.
² Publication bias suspected based on funnel plot asymmetry and significant Egger's test (p = 0.002).
³ Serious imprecision: relatively few studies and wide confidence intervals.
⁴ Serious risk of bias: single observational study with limited adjustment for confounding.
⁵ Serious imprecision: wide confidence interval (OR 3.42, 95% CI 1.67–7.01).
⁶ Very serious imprecision: very wide confidence interval crossing null value (OR 2.15, 95% CI 0.89–5.18).

## Supplementary Figure S1: Funnel Plots for Assessment of Publication Bias

**Panel A:** MRSA prevalence (19 studies). Visual interpretation shows asymmetry with a relative absence of studies reporting low MRSA prevalence with high precision. Egger's test p = 0.002, suggesting possible publication bias or small-study effects.

**Panel B:** MDR prevalence (11 studies). Visual interpretation shows a relatively symmetrical distribution. Egger's test p = 0.715, indicating no strong evidence of publication bias.

Funnel plots for assessment of publication bias. (A) MRSA prevalence (19 studies); Egger's test p = 0.002. (B) MDR prevalence (11 studies); Egger's test p = 0.715.
